# Macroautophagy and Chaperone-Mediated Autophagy in Heart Failure: The Known and the Unknown

**DOI:** 10.1155/2018/8602041

**Published:** 2018-01-18

**Authors:** Rajeshwary Ghosh, J. Scott Pattison

**Affiliations:** Division of Basic Biomedical Sciences, University of South Dakota Sanford School of Medicine, Vermillion, SD, USA

## Abstract

Cardiac diseases including hypertrophic and ischemic cardiomyopathies are increasingly being reported to accumulate misfolded proteins and damaged organelles. These findings have led to an increasing interest in protein degradation pathways, like autophagy, which are essential not only for normal protein turnover but also in the removal of misfolded and damaged proteins. Emerging evidence suggests a previously unprecedented role for autophagic processes in cardiac physiology and pathology. This review focuses on the major types of autophagic processes, the genes and protein complexes involved, and their regulation. It discusses the key similarities and differences between macroautophagy, chaperone-mediated autophagy, and selective mitophagy structures and functions. The genetic models available to study loss and gain of macroautophagy, mitophagy, and CMA are discussed. It defines the markers of autophagic processes, methods for measuring autophagic activities, and their interpretations. This review then summarizes the major studies of autophagy in the heart and their contribution to cardiac pathology. Some reports suggest macroautophagy imparts cardioprotection from heart failure pathology. Meanwhile, other studies find macroautophagy activation may be detrimental in cardiac pathology. An improved understanding of autophagic processes and their regulation may lead to a new genre of treatments for cardiac diseases.

## 1. Introduction

Recent studies show that impaired autophagy plays an important role in the progression of various forms of heart failure [1–4]. Over the past few decades, protein degradation pathways have emerged as mechanisms enabling the cells to eliminate redundant and damaged proteins. Mechanisms of protein degradation are impaired in many diseases including heart failure and contribute to their pathological progression [5, 6]. While significant advances have been made in understanding the underlying cellular and molecular mechanisms associated with heart failure, new and more effective strategies are needed. Modulating mechanisms of protein degradation may offer a promising new direction for the treatment of a host of cardiac diseases.

Tight regulation of protein turnover is essential for maintaining cellular homeostasis and survival [7]. There are two major intracellular protein degradation pathways, the ubiquitin proteasome system and autophagy-lysosomal system. In this review, we will focus on autophagic processes since the field of autophagy is relatively new and less studied, especially under pathological conditions. Autophagy is an important proteolytic mechanism that regulates the homeostasis of long-lived proteins, macromolecules including lipids, and cell organelles. Mechanisms of autophagy transport their intracellular cargo to the lysosomes for bulk degradation and recycling of macromolecules [7]. Understanding the mechanisms of protein quality control in the heart may reveal novel therapeutic avenues to treat a host of diseases including cardiac disease, the leading cause of death worldwide [8]. The goal of this review is to (i) describe the different forms of autophagy, (ii) discuss the mechanisms regulating the different forms autophagy, (iii) summarize the genetic tools available for studying autophagy, (iv) define the methods to measure autophagy function, and (v) discuss the involvement of autophagy in cardiac pathology.

## 2. Different Forms of Autophagy

**Figure 1:**
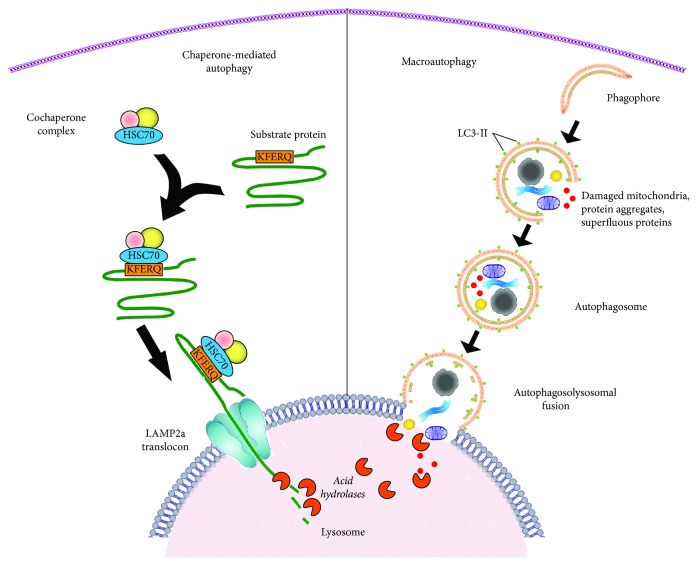
Schematic representation of the different steps of macroautophagy and chaperone-mediated autophagy. Left: chaperone-mediated autophagy targets individual proteins with a CMA-targeting (KFERQ) recognition sequence. The KFERQ sequence is recognized by an HSC70 chaperone protein with a cochaperone complex which binds the protein substrate by its KFERQ-like motif and delivers it to a LAMP2a receptor on the lysosome. The LAMP2a forms a translocation complex, which binds and internalizes the targeted protein for degradation. The acidic hydrolases in the lumen of the lysosome then lyse the protein. Right: mammalian macroautophagy begins with vesicle nucleation leading to the formation of an isolation membrane. Vesicle expansion is carried out by the coordinated action of the autophagy core machinery proteins resulting in the formation of autophagosome. Autophagosomes can engulf entire organelles like damaged mitochondria, peroxisomes, and large cargo proteins. Elongation and maturation of autophagosome membranes are discriminated by their decoration with the LC3-II protein. Mature autophagosomes then fuse with lysosomes forming autolysosomes. Finally, the sequestered materials of the autolysosomes are degraded by the acidic hydrolases of the lysosomes.

The term autophagy (auto: self; phagein: eating) was coined by Christian De Duve based on his observations of autophagic vacuole formation in rat livers perfused with the hormone glucagon [9]. Autophagy is a ubiquitous process which is responsible for eliminating harmful protein aggregates, intracellular pathogens, and superfluous proteins by the lysosomes. As more models of disease have been studied, many have observed dysregulation of autophagic processes. There are three major types of autophagy: macroautophagy, microautophagy, and chaperone-mediated autophagy. Macroautophagy employs double-membraned vesicles, known as autophagosomes, to engulf cytoplasmic proteins and organelles for delivery to the lysosome for degradation. Autophagosomes travel along microtubules and then fuse with lysosomes which are then termed autophagolysosomes [10]. Following fusion with lysosomes, the cargo delivered is degraded by lysosomal enzymes [11] (Figure 1). The byproducts of lysosomal degradation including amino acids are recycled and utilized for protein synthesis enabling salvage of energy normally used in de novo synthesis. Thus autophagy contributes to the maintenance of cellular energetic balance and quality control.

Some macroautophagic processes are believed to be more selective in the specific degradation of damaged organelles like mitochondria and peroxisomes [12, 13]. Microautophagy on the contrary does not require autophagosomes but instead involves the direct engulfment of the cargo by the invagination of the lysosomal membrane [14]. A third type of autophagy known as chaperone-mediated autophagy (CMA) is unique to mammalian cells [15]. CMA employs a cochaperone complex led by heat shock cognate 70 (HSC70) that selectively binds to cytosolic proteins containing a KFERQ-like pentapeptide sequence (Figure 1). Chaperone-bound proteins are then transported to lysosomes, where they are recognized by the lysosome-associated membrane protein type 2a (LAMP2a) receptor. LAMP2a proteins then oligomerize and form a translocon complex for internalization and degradation of chaperone-delivered cargo (Figure 1) [16–18]. A key difference between CMA and macroautophagy is that CMA delivers individual proteins for lysosomal degradation one at a time. Conversely, in macroautophagy, autophagosomes engulf and deliver predominantly larger structures for bulk degradation of cargo (Figure 1).

### 2.1. Macroautophagy

#### 2.1.1. Machinery

Autophagy is an evolutionarily conserved process occurring in all eukaryotes but is not found in prokaryotes. Studies in yeast genetics have contributed most of the knowledge about macroautophagy molecular mechanisms [19–21]. Over 30 autophagy-related genes (*ATG*) genes have been found by screening yeast mutants [22–24], and many orthologs of *ATG* genes have been identified in higher eukaryotes (Table 1) [25]. Detailed analyses have revealed several autophagic protein complexes that are involved in autophagosome formation. Suzuki et al. have identified six functional groups of Atg proteins in the yeast cells: Atg1 protein kinase complex, Atg8, Atg2-Atg18, Atg16-Atg5-Atg12 complex, Atg14 phosphatidylionositol 3-kinase complex (PtdIns3K), and Atg9 [26]. These complexes participate in steps of autophagosome formation defined as vesicle activation, nucleation, elongation, and maturation.

**Figure 2:**
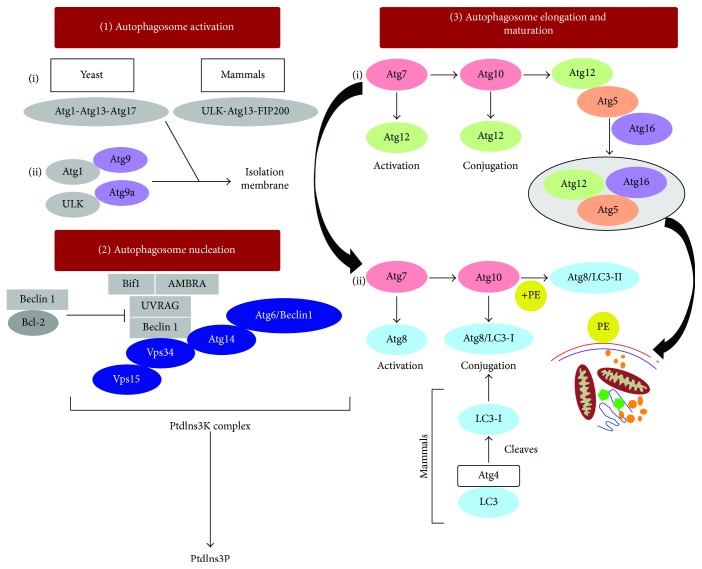
Figure depicting the various autophagy proteins involved at different stages of autophagosome formation. In yeast, macroautophagy is initiated by the formation of an Atg1 protein kinase complex which allows the recruitment of Atg9 and activation of other autophagy proteins required for autophagosome formation. This is followed by the vesicle nucleation which requires the activation of class III PtdIns3K complex leading to the formation of PtdIns3P. PtdIns3P is responsible for recruiting of effector molecules to the preautophagosomal structure needed for autophagosome formation. Autophagosome elongation and maturation are carried out by two ubiquitin-like conjugation systems: Atg12-Atg5-Atg16 and Atg8/LC3-II. LC3-II participates in vesicle elongation and substrate selection.

*(1) Vesicle Activation*. In yeast, autophagosome biogenesis is initiated by the Atg1 protein kinase complex which is linked to Atg13 and Atg17 (Atg1-Atg13-Atg17 complex) (Figure 2) [27, 28]. The mammalian counterpart of this complex is ULK-ATG13-FIP200 (200 kDa focal adhesion kinase family-interacting protein) [29, 30]. ULK (UNC-51-like kinase) is the homologue of yeast Atg1 in mammals and exists as two isoforms: ULK1 and ULK2 [31, 32]. Knockdown of ULK1 but not ULK2 inhibited macroautophagy suggesting that of the two isoforms, ULK1 is functional in mammalian cells [31]. FIP200 was shown to be an interacting partner of ULKs in mammals which contributes to the stability and kinase activity of ULK1 and was identified as the functional homologue of Atg17 [32]. Upon Atg1 complex activation, Atg9 is stimulated to recruit Atg9 vesicles to the phagophore assembly site to begin autophagosome nucleation.

*(2) Autophagosome Nucleation*. Nucleation is the first step of autophagosome formation. Activation of the class III PtdIns3K complex contributes to the vesicle nucleation during autophagosome biogenesis. This complex is comprised of Atg6 (Beclin 1 in mammalian cells), Atg14 (mATG14 in mammalian cells), PtdIns3K vacuolar protein sorting 34, and 15 (Vps34 and Vps15, also called PIK3C3) [33] (Figure 2). The activation of Vps34 is important for autophagosome formation which generates a complex consisting of Vps15, Beclin 1, AMBRA1 (activating molecule in Beclin 1-regulated autophagy protein 1), UVRAG (ultraviolet irradiation resistance-associated gene), and BIF-1 (Bax-interacting factor-1). Dissociation of Beclin 1 from its interaction partner, Bcl-2 (B-cell lymphoma/leukemia-2), an antiapoptotic protein, is an important step for the activation of Vps34. Under normal physiological (nutrient rich) conditions, Bcl-2 inhibits macroautophagy induction by binding to the BH3 domain of Beclin 1 [34]. During starvation, Beclin 1 dissociates from Bcl-2 either by (i) phosphorylation of Bcl-2 or Beclin 1, (ii) competitive displacement of Beclin 1 BH3 domain by other Bcl-2 family proteins (tBid, Bad, and BNIP3), or (iii) displacement of Bcl-2 by other Beclin 1-binding proteins (HMGB1, UVRAG, or Atg14L) [34]. This process promotes the catalytic activity of Vps34 and formation of phosphatidylinositol 3-phosphate (PtdIns3P) [34]. In yeast, PtdIns3P formation is thought to mediate the recruitment of effector molecules to the preautophagosomal structure needed for autophagosome formation [33, 35]. Activation of macroautophagy is followed by the elongation of membrane that develops into an autophagosome.

*(3) Autophagosome Elongation and Maturation*. For vesicle elongation, two ubiquitin-like conjugation systems are required in both yeast and mammals [36–38]. Herein, Atg7 acts as an E1-like activating enzyme and activates Atg12 which is then transferred to Atg10 (a homolog of E2-like conjugating enzyme) leading to the binding of Atg12 to the lysine residue of Atg5. The Atg12-Atg5 conjugate interacts with Atg16, and the resulting Atg12-Atg5-Atg16 complex attaches to the phagophore [39] (Figure 2). The second conjugation system begins by the activation of Atg8 with an exposed C-terminal lysine residue by a cysteine protease Atg4. Cleaved Atg8 is then transferred to Atg3 (an E2-like homolog) along with Atg7 which adds a phosphatidylethanolamine (PE) group to Atg8, which is also facilitated by Atg12-Atg5-Atg16 complex which behaves as an E3-like ligase [40, 41]. The mammalian homolog of Atg8, microtubule-associated protein 1 light chain (LC3), exists as LC3-I and LC3-II [42]. LC3-I is the cleaved form of LC3 generated by ATG4, and LC3-II is the lipidated form similar to Atg8 bound to PE. LC3-II participates in vesicle elongation as well as substrate selection. LC3-II level has been shown to correlate with autophagosome formation and has been used as a marker of autophagy [42, 43]. Following the elongation process, mature autophagosomes fuse with late endosomes to form amphisomes and then fuse with lysosomes, called autolysosomes [44]. Recent studies have identified several other factors associated with autophagosome maturation like UVRAG, RUBICON, and valosin-containing protein (VCP) [45–48]. UVRAG binds to the homotypic fusion and vacuole protein sorting (HOPS) complex and activates RAB7 thereby promoting autophagosome fusion with late endosomes and lysosomes [45]. It is thought that Atg8/LC3-II is delipidated by Atg4 and that the freed Atg8/LC3 is recycled for autophagosome biogenesis [49, 50] (Figure 2).

Although autophagy is a highly conserved process in both yeast and mammals, there are some differences in the core processes involving autophagosome formation [33]. One study showed different distribution of PtdIns3P which may account for differences in autophagic processes in yeast and mammals [51]. Additionally, several *ATG* genes found in yeast have yet to have homologous mammalian genes identified (Table 1). Moreover, while yeast have single Atg 4, 8, and 18 proteins, mammals have multiple homologs for ATG 4, 8, and 18 proteins which may lead to differences in the macroautophagic processes in yeast and mammals.

#### 2.1.2. Regulation of Macroautophagy

A large number of signaling molecules and pathways have been identified which regulate cell survival and autophagy. Understanding these complex pathways is becoming increasingly important as new functions of macroautophagy in pathological conditions are revealed. In mammals, the highly conserved kinase, mTOR, is known to regulate various cell signaling pathways in response to several factors including amino acids, stress, oxygen, energy levels, and growth factors which are critical for maintaining cellular homeostasis [52]. Of the two functional forms of mTOR, complex 1 (mTORC1) has been reported to play a key role in the regulation of macroautophagy by its direct interaction with ULK1-ATG13-FIP200 trimeric complex [53, 54]. Phosphorylation of ATG13 by mTOR is important for the activation of ULK1 and cytoplasm to vacuole targeting (Cvt) [28]. Activation of macroautophagy takes place by the inhibition of mTOR which in turn activates ULK1 under starved conditions [53, 55]. Another study reported that ATG14-containing PIK3C3 is specifically regulated by mTORC1 in response to amino acid starvation [56]. Additionally, the same group studied the contribution of AMP-activated protein kinase (AMPK) in the regulation of macroautophagy in amino acid-starved cells. Similar activation of Atg14-containing PIK3C3 was observed in wild-type and knockout models of AMPK in MEFs [56]. Under nutrient-rich condition, the activation of mTORC1 negatively regulates macroautophagy by the phosphorylation of the kinase ULK1 at the Ser757 and prevents its interaction with the AMPK thereby inhibiting macroautophagy induction [53, 57, 58]. Upon energy starvation, AMPK differentially regulates various VPS34 complexes [59]. AMPK was shown to inhibit nonautophagy VPS34 complexes and to activate the proautophagy VPS34 by the phosphorylation of Beclin 1 [59].

Death-associated protein kinase (DAPK) is a Ser/Thr kinase that is regulated by Ca^2+^/calmodulin and has been identified as a critical regulator of macroautophagy [60]. DAPK expression has been shown to correlate with increased autophagosome formation, confirmed by the enhanced accumulation of the macroautophagy marker, LC3 [61, 62]. DAPK can stimulate macroautophagy by phosphorylating Beclin 1 at Thr119 thereby preventing its association with Bcl-2 [62, 63]. It was shown that DAPK can activate macroautophagy through the phosphorylation of protein kinase D (PKD) which in turn phosphorylates and activates VPS34 [64]. Increased accumulation of the lipidated form of LC3 has been observed with oxidative stress. It was shown that suppressing DAPK levels by shRNA decreased the LC3-II/LC3-I ratio interpreted as inhibiting oxidative stress-induced macroautophagy [64].

#### 2.1.3. Genetic Models Studying Macroautophagy in Heart Failure

Various genetic models to study the physiological and pathological significance of macroautophagy in the heart have been developed. Gain and loss of function studies have defined the functional role of macroautophagy in cardiac pathology. Studies using Atg genes to study macroautophagy function in cardiac pathology are summarized in Table 1. Cardiac-specific deletion of *Atg5* in adult mice exhibited cardiomyopathy characterized by cardiac hypertrophy, contractile dysfunction, and left ventricular dilation as well as increased accumulation of ubiquitinated proteins [65]. The same group showed that during early cardiogenesis, suppressing *Atg5* did not induce any cardiac abnormality suggesting the occurrence of compensatory mechanisms to antagonize the adverse effects of macroautophagy inhibition. However, *Atg5*-deficient mice subjected to pressure-overload stress demonstrated increased LC3-II levels during heart failure at 4 weeks. Expression of mCherry-*atg5^K130R^*, a dominant negative mutant of *Atg5*, in HL-1 cardiomyocytes was shown to decrease numbers of GFP-LC3 vacuoles following ischemia-reperfusion (I/R) [66].

Gain of Atg7 in cultured cardiomyocytes expressing *CryAB^R120G^* showed reduced accumulation of misfolded proteins. Depleting *Atg7* in *CryAB^R120G^*-expressing cardiomyocytes increased the pathology [67]. Flux assays indicated by increased LC3-II levels in the presence of Bafilomycin A_1_ revealed that enhancing ATG7 levels in *CryAB^R120G^-*expressing cells could rescue *CryAB^R120G^*-induced macroautophagy inhibition [67]. This study was extended into mouse models of cardiomyopathy. Mice expressing the mutant *CryAB^R120G^*, a proteotoxic model of desmin-related cardiomyopathy, are characterized by increased accumulation of misfolded protein aggregates in the hearts. Reduced autophagic activity was shown in the hearts of mutant *CryAB^R120G^* both by ultrastructural analysis and decreased autophagic flux in *CryAB^R120G^-*expressing cardiomyocytes [67, 68]. Transgenic mice overexpressing ATG7 displayed normal cardiac function and cardiac macroautophagy as demonstrated by increased levels of LC3-II. Additionally, ultrastructural studies showed an increased number of autophagic structures like amphisomes. When *Atg7* was coexpressed in the mutant *CryAB^R120G^*-expressing mice, cardiac autophagic function improved as demonstrated by upregulated LC3-II turnover in the presence of chloroquine and increased number of autolysosomes. Induction of macroautophagy in the *CryAB^R120G^* mice was associated with reduced pathology indicated by reduced protein aggregate formation [2]. In another study, cardiac-specific deletion of Atg7 showed decreased LC3-II and increased p62 accumulation post-I/R treatment [69]. Impaired macroautophagic function coincided with further aggravation of myocardial I/R injury including cardiac hypertrophy, contractile dysfunction, and severe cardiac fibrosis with accumulation of a negative cytoskeleton organization regulator, CLP36.

*Beclin 1*-deficient mice were generated to study the effect of macroautophagy in diabetes-induced cardiac dysfunction [70]. In wild-type mice, suppression of *Beclin 1* expression did not interfere with LC3-II levels in the presence and absence of a lysosomal inhibitor, Bafilomycin A_1_. *Beclin 1* deletion improved cardiac function in streptozotocin diabetic mice accompanied by decreased apoptotic cell death. These results were further confirmed in the *ATG16L1*-deficient mice where improved cardiac function was observed in diabetic hearts. Streptozotocin-induced diabetic mice exhibited lower levels of LC3-II. Dual deletion of both *Beclin 1* and *ATG16L1* further impaired macroautophagy in the diabetic hearts. Furthermore, transgenic mice expressing *Beclin 1* exacerbated diabetic cardiac injury compared to the wild-type diabetic mice [70]. The authors interpreted the reduced macroautophagy as a protective mechanism to improve diabetic-induced cardiac dysfunction. Consistently, another study showed that *Beclin 1* deletion in the heart reduces hemodynamic stress-induced macroautophagy in the heart which is associated with reduced pathological remodeling. On the contrary, *Beclin 1* overexpression in the hearts of the mice subjected to pressure-overload stress exhibited pathological cell growth and increased GFP-LC3 levels [71]. Following I/R, GFP-LC3/*Beclin 1*^+/−^ mice demonstrated significant reduction in the number of GFP-LC3 puncta compared to the wild-type mice. Consistent with the other studies, cardiac injury was substantially reduced in the *Beclin 1*-null mice [72].

In a recent study, cardiac-specific deletion of *Ulk1* in mice exhibited increased lipoprotein lipase levels. Under high-fat diet conditions, *Ulk1*-deleted mice showed further exacerbation of lipotoxicity associated with retarded cardiac function [73]. An important component of ULK1 complex, FIP200, is essential for macroautophagy initiation [32]. *FIP200* knockout in mice led to embryonic death associated with heart failure. *FIP200* knockout embryos demonstrated severe cardiac abnormalities including thinning of the ventricular wall and ventricular dilation with sparsely cellular myocardium [74]. Similarly, *Atg13*-deficient mice generated by using the clustered regularly interspaced short palindromic repeat (CRISPR)/Cas9 system showed myocardial growth defects which included thinning of the ventricular wall [75]. Both *FIP200* and *Atg13* were found to be necessary for cardiac development during embryogenesis [74, 75].

Manipulation of macroautophagy genes has helped to gain a better understanding of the role and contribution of macroautophagy to cardiac pathology. Similar genetic approaches can be employed to study the functional significance of other ATG proteins like the WIPI members, SNX30, SNX4, and ATG101 (Table 1). These approaches may lead to the identification of new therapeutic targets for the treatment of cardiac pathology. However, far more loss of macroautophagy gene studies has been published than gain of macroautophagy studies relative to cardiac physiology and pathology.

### 2.2. Chaperone-Mediated Autophagy

#### 2.2.1. Machinery

One of the distinctive features of chaperone-mediated autophagy (CMA) as opposed to macroautophagy is its highly selective nature of recognizing and degrading individual cytosolic proteins [76, 77]. CMA specifically degrades the target proteins bearing a unique recognition pentapeptide motif (KFERQ-like) [78]. Another unique aspect is the direct translocation of the substrate proteins into the lysosomal lumen via a unique LAMP2a receptor, without the need of any cytosolic vesicles (autophagosomes) [78–80]. Cytosolic chaperones like the heat shock cognate protein of 70 kDa (HSC70) participate in the recognition, unfolding, and translocation of substrates bearing the KFERQ-like motif [15]. The translocation complex comprised of LAMP2a and a luminal HSC70 within the lysosomes (lys-HSC70) forms an integral part of the CMA machinery [81, 82]. Studies have shown that the occurrence of lys-HSC70 is essential for CMA activity, the lack of which inhibits CMA function [79, 83]. Cytosolic HSC70 chaperone protein which binds the substrate by its pentapeptide motif transfers it to the monomeric form of LAMP2a receptor [84]. Multimerization of LAMP2a into a 700 kDa protein complex is achieved by the binding of the substrates which is necessary for the subsequent internalization and degradation of the proteins in the presence of lys-HSC70 [84]. CMA is thought to have two major functions: maintenance of cellular quality control by degrading damaged proteins and maintenance of cellular energetic homeostasis by recycling the amino acids resulting from the degradation of superfluous proteins [15].

#### 2.2.2. Regulation of CMA

Under basal conditions, CMA activity has been detected in various tissues like the liver, kidneys, brain, and spleen [85–89]. CMA activity can also be upregulated under various stress conditions. It has been shown that similar to macroautophagy, nutritional stress can lead to increased CMA activity which is associated with increased levels of both lys-HSC70 and LAMP2a [17, 83, 87]. Both macroautophagy and CMA can be upregulated by nutritional stress. However, macroautophagy is activated within 4–6 h of starvation which then gradually decreases. The decrease in macroautophagy coincides with increased CMA activation which occurs only after prolonged starvation with maximal activity achieved at 24 h [90]. It was suggested that ketone bodies generated during prolonged starvation may provide the required signal for CMA activation [91]. The rate-limiting step of CMA activity is the binding of the substrates to LAMP2a, and therefore changes in LAMP2a levels regulate CMA activity [17, 80, 92]. In addition to starvation, oxidative stress has been shown to upregulate CMA function [93]. It was shown that CMA competent lysosomes could degrade oxidized proteins through enhanced CMA activity in the rat liver [93]. However, in aged animals, the ability of CMA to remove the oxidized proteins was significantly reduced suggesting that aging negatively regulates CMA function. Studies suggest that CMA activity can be regulated by cytosolic levels of GTP through the interaction of LAMP2a with two proteins, GFAP and EF1*α* [94]. The stability of the multimeric LAMP2a translocation complex is achieved by GFAP binding which antagonizes the disassembling property of HSC70 [94]. Several studies have shown the involvement of CMA in neurodegenerative diseases and cancer [89, 95]. Although CMA activity has been reported in the hearts of fasted animals [78, 96], the causal role of CMA in the heart under physiological and pathological conditions remains to be defined.

### 2.3. Mitophagy

Cells rely on mitochondrial intracellular ATP from oxidative phosphorylation [97]. Therefore, it is not surprising that impaired mitochondrial function has pathological consequences. Mitochondrial dysfunction is closely associated with cardiovascular diseases, diabetes, cancer, and neurological diseases [98–101]. Malfunctioning mitochondria may occur due to impaired electron transport chain function resulting in decreased ATP synthesis, insufficient substrates for oxidative phosphorylation, or from overproduction of reactive oxygen species. Therefore, it is important for cells to remove damaged mitochondria for normal cellular function. Cells maintain quality control by degrading damaged mitochondria by a process known as mitophagy. Mitophagy is a selective form of macroautophagy wherein the autophagosomes selectively target dysfunctional mitochondria for their degradation and thereby maintain proper cellular homeostasis. Disruption in the mitochondrial membrane potential can trigger the clearance of dysfunctional mitochondria in both yeast and mammals [102, 103]. Additionally, mutations in the mitochondrial DNA can lead to organelle degradation by mitophagy [104].

Genome-wide screening of mitophagy-deficient yeast mutants led to the identification of Atg32 which plays a primary role in the selective degradation of mitochondria by autophagy and acts as a mitophagy receptor [105, 106]. Deletion of Atg32 gene resulted in the loss of mitophagy with no changes in bulk macroautophagy or the cytoplasm to vacuole targeting (Cvt) pathway [105, 106]. Another pathway related to the selectivity of autophagosomes for damaged mitochondria involves the mitochondrial proteins PINK1 and its E3 ligase PARKIN [107]. Under normal conditions, PINK1 is degraded rapidly; however, it stabilizes on damaged or depolarized mitochondria which facilitates the activation of PARKIN [108–110]. The E3 ligase activity of PARKIN also ubiquitinates several other mitochondrial proteins including MFN1 and 2, VDAC1, TOMs, BAK, and MIRO which may facilitate turnover of defective mitochondria [111–113]. The autophagic adaptor protein, p62/SQSTM1/sequestosome-1 which contains a LC3-interacting domain, also interacts with Parkin which may facilitate the recruitment of damaged mitochondria to the autophagosome by binding to LC3-II [114]. Apart from the PINK1-PARKIN signaling pathway, several other regulators like the mitochondrial membrane protein FUNDC1, proapoptotic proteins like NIX and BNIP3, and the macroautophagy proteins, ULK1 and ATG7, have also been suggested to mediate mitochondrial degradation reviewed by Ding and Yin [115].

### 2.4. Techniques to Monitor Different Forms of Autophagy

#### 2.4.1. Methods to Assess Macroautophagy

Several reviews have discussed the methods and assays to monitor autophagy [116–118]. The most traditional method for analyzing the ultrastructural morphology of autophagic structures is transmission electron microscopy (TEM). TEM allows one to observe autophagosomes at different stages of maturation, sequestered autophagosomal cargo, and their localization with other cellular organelles: lysosomes, mitochondria, ER, and so forth, to yield a qualitative representation of autophagy [119]. Early macroautophagic compartments appear to contain cytoplasmic content or organelles while matured autophagosomes that have fused with lysosomes contain partially degraded cytoplasmic content or organelles. It has been argued that while TEM offers certain advantages, it does not require antibodies or probes; using this technique requires specialized skills. Further, analysis of TEM images requires expertise to accurately define the ultrastructural features observed, the lack of which leads to erroneous data interpretation.

Several techniques have been developed to monitor autophagosome number or content, using the autophagosome marker protein LC3-II. Antibodies specific for LC3 can be used for immunostaining or to determine LC3-II protein levels by immunoblotting. Fluorescent microscopy coupled with overexpression of a GFP-LC3 reporter is a technique used to visualize and quantify autophagosomes. This technique allows one to visualize the LC3-positive punctate structures that represent autophagosome numbers [120].

Early macroautophagy studies suggested that increased autophagosome numbers or levels (evidenced by changes in GFP-LC3 or LC3-II levels) corresponded to increased autophagic activity. However, determining the number of autophagosomes is not an indicator of cellular macroautophagy activity. Autophagosome levels can be increased due to increased autophagosome synthesis or from impaired lysosomal degradation. Another reporter, GFP, fused to Atg5 (GFP-Atg5) has been utilized to label early stage autophagosomes [121]. One of the disadvantages of GFP-LC3 is that GFP-fusion proteins are sensitive to lysosomal proteases resulting in loss of florescence upon fusion with lysosomes. To address this issue, a tandem fluorescent-tagged LC3 (tfLC3) expression vector was developed which allows analysis of autophagosomes versus autolysosomes. The construct consists of RFP-GFP-LC3 fusion where RFP (red fluorescent protein) is resistant to the acid hydrolase degradation and thus maintains fluorescence [120]. It should be noted that this assay measures the fusion of lysosomes with autophagosomes and may not be an indicative of cargo degradation and thus does not represent a true autophagic flux assay. A well-known substrate of macroautophagy, p62, binds directly to LC3 and gets efficiently degraded by the lysosome [122]. Increased or decreased p62 protein levels are associated with increased autophagic activity. However, p62 may also be degraded by the ubiquitin-proteasome system and p62 levels are increased by a host of cellular stress and signaling pathways [123]. Thus changes in p62 levels are only a corroborating measure for macroautophagy analysis, not proof of altered macroautophagy activity. Based on the limitations of many assays, it is suggested that a combination of different assays be utilized.

Currently autophagic flux assays are the gold standard method for assessing alterations in macroautophagic activity. During macroautophagy, LC3 is converted to LC3-I and then processed into LC3-II. Only mature LC3-II decorates autophagosome membranes [42]. The assay measures the relative turnover of LC3-II in the presence and absence of lysosomal inhibitors like pepstatin A, chloroquine, ammonium chloride, or Bafilomycin A_1_. Lysosomal inhibition impairs the formation of an autolysosome which results in increased accumulation of LC3-II and other substrates degraded by the lysosome [124]. By clamping autophagosomal degradation, any changes in autophagosome levels must be due to differences in autophagosome synthesis.

*In vivo* models for studying macroautophagy have also been developed. Systemic GFP-LC3 transgenic mice have been generated for determining autophagosome level changes [125]. In the field of cardiac pathology, cardiac-specific expressing mCherry-LC3 transgenic mice have also been generated to study autophagosomal changes, which have been used in mice subjected to I/R injury and ischemic preconditioning [126–128]. Monitoring macroautophagy using flux assays in the presence of lysosomal inhibitors has been shown *in vivo* [129–131]. However, various factors like the concentration of the inhibitor and the duration of treatment to achieve effective inhibition must be carefully considered. To observe greater changes in LC3-II levels, mice are often subjected to starvation to induce macroautophagy [129, 131]. Analyzing macroautophagy *in vivo* requires more empirical optimization, and each method should be carefully validated to obtain reliable results.

#### 2.4.2. Pharmacological Inhibitors and Activators of Macroautophagy

Various pharmacological inhibitors and activators of macroautophagy have been identified which has been reviewed by Vakifahmetoglu-Norberg et al. [132]. PI3-kinase inhibitors such as wortmannin/LY294002 and 3-methyladenine (3-MA) and VPS34 kinase inhibitor like SAR205 among others may be used to inhibit macroautophagy (primarily under starvation conditions). 3-MA has been shown to inhibit autophagosome formation [133], wortmannin/LY294002 is the inhibitor of autophagosome formation, and SAR205 disrupts the late endosome and lysosome compartments preventing autophagy [134]. The final step of macroautophagy (degradation of autophagic cargo in the autolysosome) can be inhibited by ammonium chloride, chloroquine (lysososmotropic agent), Bafilomycin A_1_, and lysosomal protease inhibitors such as E64d and pepstatin A. Ammonium chloride, chloroquine, and Bafilomycin A_1_ act by neutralizing the lysosomal acidic compartments thereby inhibiting the fusion of autophagosomes and degradation of cargo proteins. E64d and pepstatin A inhibit lysosomal proteases and thereby blocking lysosomal protein degradation. E64d is an aspartic protease inhibitor, and pepstatin A is a cysteine, serine, and threonine protease inhibitor [135]. As with most pharmacological agents, a major concern with employing these inhibitors is that they are not macroautophagy-specific [135]. They inhibit other processes which require lysosomal function/degradation, including endosomal turnover and CMA.

Activators of macroautophagy include rapamycin and Torin1 which inhibit mTOR have been shown to activate macroautophagy [132]. The mechanism of rapamycin-mediated macroautophagy activation involves the formation of a complex with FK506-binding protein (FKBP12), and the complex binds to and inhibits mTOR kinase activity thereby activating macroautophagy [136]. Rapamycin is shown to activate Atg1 and increase the binding affinity of Atg1 to Atg13 and Atg17 in yeast [28]. Torin1 is another highly selective ATP-competitive inhibitor of mTOR complexes. It blocks phosphorylation of all mTORC1 substrates and is a more potent inducer of macroautophagy compared to rapamycin. Other pharmacological activators include Metformin, and a fusion peptide, Tat-Beclin 1, which was shown to activate macroautophagy [137, 138]. Metformin activates macroautophagy by the phosphorylation and activation of AMPK [139]. The Tat-Beclin 1 peptide functions by competing with a Beclin 1-negative regulator protein, named GAPR 1, thereby inducing macroautophagy [137].

Pharmacological agents may interfere with other cellular processes other than macroautophagy, and it is therefore recommended to confirm findings with other genetic or pharmacologic approaches and flux assays to accurately determine macroautophagy function both *in vitro* and *in vivo*. Before manipulating macroautophagy for the treatment of cardiovascular disease, the results from the published papers must be carefully interpreted to determine the effect of macroautophagy activation and inhibition in response to cardiac pathology.

#### 2.4.3. Methods to Assess CMA Function

The assays to monitor CMA function in cells and tissues have been described by Kaushik and Cuervo [85]. One of the most efficient and reliable ways to determine CMA activity is by measuring LAMP2a levels in cultured cells or tissues. HSC70 is the cytosolic chaperone that targets the CMA substrate proteins to the lysosomes. HSC70 is expressed constitutively under normal conditions. Increased CMA activity positively correlates with increased lysosomal LAMP2a and lysosomal HSC70 levels [83, 92]. Total LAMP2a and HSC70 levels can be detected by immunoblot analysis as markers of CMA. Studies related to gain and loss of CMA function by overexpressing or silencing LAMP2a and knockdown of HSC70 can define the causal roles of CMA in different cells and tissues under pathophysiological conditions. However, lysosomal levels of LAMP2a and HSC70 occur only in a subset of lysosomes which are competent for CMA. Under certain conditions, total cellular levels of LAMP2a differ from LAMP2a/HSC70 levels in purified lysosomal fractions isolated from cells/tissues. CMA function can be inferred by colocalization of HSC70 with LAMP2a [92].

Another assay to determine CMA function is to measure the translocation and degradation of CMA substrates in purified CMA-competent lysosomes from cells/tissues of interest [79]. Protein degradation studies can be performed in isolated lysosomes where labeled substrate turnover can be determined by incubating intact lysosomes with CMA substrates in the absence and presence of exogenous lysosomal protease inhibitors. CMA substrate proteins can then be immunoblotted and quantified to define CMA-specific lysosomal. Another assay to measure CMA substrate delivery and degradation can be performed by employing fluorescent CMA reporters [140]. A photoswitchable fluorescent KFERQ-PS-CFP2 reporter can be used to determine lysosomal internalization and degradation of the reporter protein with different excitation and emission spectra in cells as described previously [140]. Changes in fluorescence is measured which gives an indication of CMA activity.

The methods described for monitoring CMA function *in vivo* studies are limited. However, both gain- and loss-of-function mouse models have been developed for LAMP2a which is both necessary and sufficient to control CMA activity [86, 141]. Inducible, conditional transgenic mice (Alb-Tet-off-L2A) where an exogenous copy of LAMP2a can be regulated by doxycycline have been generated to study increased CMA function in the liver [86]. The Cre-*loxP* system was also used to generate conditional LAMP2a knockout mice, which have been used to study the loss of CMA function in the liver [141]. To better understand the significance of CMA in different pathological conditions, genetically modified animals with gain or loss of CMA activity need to be used in combination with various disease conditions. The study of CMA *in vivo* is still in its infancy, and additional studies are needed to understand the breadth and depth of CMA in both basal physiological and disease conditions.

### 2.5. Macroautophagy in Different Forms of Heart Failure

Cardiovascular disease is the leading cause of mortality and accounts for one in every three deaths in the United States each year [8]. A decade ago, little was known about autophagy in the heart. Since then, the most extensively studied form of autophagy, macroautophagy, has been shown to be essential in both normal physiology and in numerous forms of cardiac pathology [66, 142, 143]. Many forms of heart failure are associated with the accumulation of aberrant proteins due to impaired protein degradation [144–147]. Under normal physiological conditions, the heart maintains basal levels of macroautophagy which is essential for maintaining cardiac homeostasis as illustrated by numerous loss of function models (Table 1). The protective function of macroautophagy is especially imperative under conditions of stress like ischemia, starvation, and *β*-adrenergic stimulation where macroautophagy mainly acts as a prosurvival mechanism by removing toxic protein aggregates and organelles [128, 148, 149].

#### 2.5.1. Macroautophagy and Cardiac Hypertrophy

Cardiac hypertrophy represents a physiological adaptation of the heart in response to increased cardiac workload as with hypertension or due to compensatory loss of contractile units with heart failure. This condition eventually leads to a pathophysiological state where there is progressive thickening of the left ventricle. Cardiomyocyte hypertrophy associated with myocardial infarction is basically an adaptive remodeling process where the body attempts to compensate for the loss of functional contractile tissue. Cardiac hypertrophy increases the risk for developing sudden cardiac failure [150]. Recently, numerous regulators of cardiac hypertrophy have been identified which correlate with macroautophagy function [146, 151–153]. Cathepsin L, a lysosomal enzyme, has been shown to play an important role in protecting myocardium via activation of autophagy in cardiac hypertrophy [146]. Cathepsin L-deficient neonatal cardiomyocytes induced by phenylephrine exhibited a more pronounced hypertrophic response compared to the wild type. Consistently, cathepsin L-deficient mice subjected to aortic banding showed exaggerated cardiac hypertrophy compared to sham animals. Interestingly, the increase in hypertrophy was accompanied by increased LC3-II/LC3-I ratio and p62 levels. Cathepsin L overexpression was able to partially improve cardiac function in pressure-load-induced hypertrophy. [146]. However, the direct effect of cathepsin L overexpression on autophagic activity was not shown.

Another protein, exchange protein directly activated by cAMP 1 (EPAC1), has been shown to increase the levels of lipidated LC3-II and Beclin 1 [152]. EPAC1-regulated signaling events include cardiac contractility and stimulation of cell growth through calcium mobilization. Interestingly, the authors reported that EPAC1-induced cardiomyocyte hypertrophy triggered the activation of macroautophagy through the Ca^2+^/CaMKK*β*/AMPK pathway [152]. *epac1* deletion resulted in decreased LC3-II and Beclin 1 levels in isoproterenol-treated *epac*-null mice compared to the wild-type animals. *epac1* suppression prevented *β*-adrenergic-induced cardiac remodeling which may suggest a detrimental role of macroautophagy stimulation in cardiac hypertrophy [152]. Conversely, another recent study reported macroautophagy as an adaptive mechanism. *Sestrin 1*, a target gene for p53 tumor suppressor, is shown to participate in mTOR inhibition. In experimental models of pressure-overload and phenylephrine-induced cardiac hypertrophy, *Sestrin 1* gene and protein expression declined which correlated with decreased macroautophagy. These results suggest that *Sestrin 1* may positively regulate macroautophagy as a mechanism of cardiac hypertrophy attenuation [153].

The pathological consequences of cardiac hypertrophy coincide with altered macroautophagy function [154, 155]. Conflicting results exist regarding the role of macroautophagy in cardiac hypertrophy [156]. While enhanced macroautophagy in hypertrophy is beneficial in some models, increased macroautophagy activation appears to be maladaptive in others [154–158]. *Beclin 1* ablation decreased markers of cardiac macroautophagy as shown by fewer GFP-LC3-II punctate structures. Additionally, *Beclin 1* deletion diminished pathological remodeling induced by pressure overload [71] suggesting a detrimental role of macroautophagy in cardiac pathology. On the other hand, autophagic response has been shown to be dependent on the stage and severity and type of cardiac hypertrophy. During the early stages of TAC-induced hypertrophy, a marker of macroautophagy, LC3-II levels were decreased. However, with increasing severity, an augmented increase in LC3-II levels was observed, suggesting that macroautophagy may be beneficial in the failing myocardium [65]. Several factors may determine whether macroautophagy is protective or maladaptive including the age, species, variability of the experimental conditions, and genes or signaling pathways manipulated. Hemodynamic stress in the heart has been shown to trigger a macroautophagy response. Increased LC3-II levels and increased numbers of autophagosomes and autolysosomes were detected in the hearts subjected to thoracic aortic banding (TAB). These results were further confirmed by expressing cardiac-specific GFP-LC3 in the animals subjected to TAB, and an increase in autophagosome numbers was detected. These findings were accompanied with increased Beclin 1 levels which resulted in pathological remodeling [71]. Targeting macroautophagy may open up new avenues for treating heart failure, but further studies are required to determine the precise mechanism(s) which result in beneficial versus detrimental macroautophagy with cardiac pathology.

#### 2.5.2. Macroautophagy in Myocardial Ischemia and Reperfusion Injury

Ischemia/reperfusion (I/R) refers to the period of interrupted blood supply to an organ or tissue (ischemia) followed by the restoration of perfusion or reoxygenation (reperfusion). Restoration of blood supply is often accompanied by increased tissue injury and cell death [159]. I/R injury is a manifest in myocardial infarction, ischemia stroke, kidney injury, and intestinal ischemia which are the leading causes of morbidity and mortality [160].

Upregulation of macroautophagy has been shown in the heart during ischemia and reperfusion [66, 72, 161, 162]. Different autophagic responses have been described when the myocardium is subjected to I/R. An earlier study showed that brief exposure to hypoxia (20 min) did not activate macroautophagy; however, reoxygenation (30 min) increased the number of autophagic vacuoles as determined by electron microscopy in rabbit hearts [163]. Increasing the time of hypoxia insult to 40 min enhanced the number of autophagosomes which further increased upon reperfusion (30 min) [163]. Autophagic markers like LC3-II and Beclin 1 were shown to increase when the isolated rat hearts were subjected to 30 min of global ischemia and 2 h of reperfusion suggesting increased autophagosome formation [149]. Consistently, depletion of *Beclin 1* levels in cardiomyocytes decreased the I/R-induced macroautophagy [162]. Macroautophagy has been shown to be either protective or harmful in the context of I/R injury. It was shown that oxidative stress induces macroautophagy during I/R in mouse hearts [164]. Increased autophagosome-lysosome fusion was observed in tfLC3 (tandem fluorescent mRFP-GFP-LC3) expressing cultured myocytes in the presence of H_2_O_2_ suggesting oxidative stress induces autophagosome degradation in isolated cardiomyocytes. The antioxidant, MPG (N-2-mercaptopropionyl glycine), completely reversed H_2_O_2_-induced changes. Similarly, in transgenic mice expressing tfLC3, I/R treatment increased fusion. MPG was also able to reduce the infarct size resulting from I/R injury [164]. The authors argued that macroautophagy induction during I/R is detrimental and contributed to overall cell death and myocardial injury.

On the contrary, another study reported the occurrence of impaired autophagic flux during I/R in neonatal cardiomyocytes which was accompanied by increased oxidative stress and cell death [126]. I/R treatment caused an impaired autophagic flux (determined by calculating the ratio of autophagosome abundance in the presence and absence of chloroquine) in mice expressing cardiac-specific GFP-LC3. Reperfusion also led to increased Beclin 1 levels and decreased lysosome-associated membrane protein-2. These changes were accompanied by increased ROS generation and cell death [126]. Another study demonstrated that overexpression of *Beclin 1* enhanced autophagic flux (GFP-LC3 levels in the presence and absence of lysosomal inhibitors) and confers protection to cardiomyocytes following I/R injury [66]. A mitochondrial proapoptotic protein, BNIP3 (a BH3 only member of the Bcl-2 family), which is expressed in the adult rat heart has been demonstrated to play a role in I/R injury [165]. It was found that BNIP3 contributes to I/R injury in the heart [165]. Expressing a mutant form of *Bnip3* (*Bnip3*Δ*TM*) in the hearts was associated with protection against I/R injury and improved cardiac function [165]. Overexpression of *Bnip3* resulted in upregulation of macroautophagy indicated by increased GFP-LC3 levels which is suggested to be a protective mechanism adapted by the heart [165].

During postinfarction left ventricular cardiac remodeling, immunofluorescent labeling showed increased levels of macroautophagy markers, LC3-II and p62, and electron microscopy revealed increased autophagosome formation in the surviving myocardium compared to the infarct region. These markers were associated with upregulation of phosphorylated AMPK levels in the myocardium which suggested a protective role of macroautophagy during postinfarction cardiac remodeling [166]. Hibernating or chronically ischemic myocardium represents viable cells but with impaired contractile function due to reduced blood flow which can be partially or completely restored by reperfusion [167, 168]. Autophagic vacuoles detected by electron microscopy were present only in the chronically ischemic region, after subjecting pig hearts to coronary stenosis. Additionally, the lysosomal protein levels and activities of Cathepsins B and D were also increased in the chronically ischemic region [161]. Consistently, an increased ratio of LC3-II/LC3-I was observed [161]. The authors interpreted these findings as activation of autophagy conferred protection to chronically ischemic myocardium against apoptosis [161]. These studies suggest that macroautophagy is upregulated in postinfarction cardiac remodeling and in the hibernating myocardium thereby antagonizing the deleterious effect of ischemia.

STAT1 activation increases in cultured cardiomyocytes in response to I/R injury. During I/R, *Stat1-*deficient heart demonstrated decreased infarct size and increased LC3-II/LC3-I ratio and Beclin 1 levels [169]. These data suggest that STAT1 activation in the heart during I/R may negatively regulate macroautophagy and prevents macroautophagy-mediated cardioprotection. Currently, it is not clear whether macroautophagy activation during I/R is a protective response adapted by the heart to neutralize cardiac pathogenesis or macroautophagy itself contributes to cardiac damage. Maintaining basal levels of macroautophagy while reducing excessive macroautophagy during I/R injury may prove to be clinically beneficial for treating I/R-induced heart failure patients.

#### 2.5.3. Macroautophagy and Proteotoxicity-Induced Cardiomyopathy

Accumulation of misfolded or aberrant proteins may serve as central triggers for inducing autophagic processes. One model of desmin-related cardiomyopathy caused by a missense mutation in the *α*B-crystallin (*CryAB^R120G^*) is characterized by increased amyloid and aggregate formation as well as attenuated macroautophagy in the failing heart [2]. *Atg7*-induced autophagic flux has been shown to reduce cardiac proteotoxicity and to prolong survival in the transgenic *CryAB^R120G^* mouse model [2]. Transgenic mice overexpressing *Myozap*, a novel cardiac-enriched intercalated disc protein, exhibit cardiomyopathy. The pathological consequences of *Myozap* overexpression involved hypertrophy of the heart and left ventricular dilation which was further exacerbated in transgenic mice subjected to pressure overload [170]. Similar to desmin-related cardiomyopathy, the *Myozap*-overexpressing hearts showed increased protein aggregate formation. Additional studies revealed elevated lipidated LC3-II levels in the *Myozap* transgenic mice indicative of increased autophagosome formation. Levels of p62 were also increased in the transgenic mice [170]. Whether activation of macroautophagy in the *Myozap* transgenic hearts is beneficial or detrimental has not been causally defined. However, other studies have identified the protective role of macroautophagy in the heart during proteotoxic stress [67, 171, 172]. Although macroautophagy is thought to play an important role in cardiac pathology, it is not yet clear what factors determine whether macroautophagy upregulation is beneficial or harmful.

#### 2.5.4. Autophagy and Hypertensive Heart Disease

Hypertension is a major risk factor for cardiovascular disease and stroke. The prevalence of hypertension is estimated to be 34% among adults in the USA [8]. Although compensatory hypertrophy counteracts the adverse effects of pressure overload, chronic hypertrophic stress eventually leads to contractile dysfunction and heart failure [173]. Nitric oxide (NO) inhibitor, N*^ω^*-nitro-l-arginine methyl ester (L-NAME), which induces hypertension is known to cause cardiac hypertrophy and fibrosis due to the activation of renin–angiotensin–aldosterone system [174, 175]. Hypertensive mouse models when challenged with L-NAME showed a decrease in LC3-II/LC3-I ratio and increase in p62 levels suggesting inhibition of macroautophagy. This was associated with decreased phosphorylation of macroautophagy signaling molecules, AMPK*α* and ULK1, and activation of mTOR which correlated with cardiac dysfunction and oxidative stress [176]. The antioxidant, metallothionein, was shown to improve cardiac function in the L-NAME-treated animals. While various studies have shown macroautophagy inhibition by NO inhibitor in the heart, it was demonstrated that NO itself impairs autophagic flux in mammalian cells [177]. Stable mRFP-GFP-LC3 expressing HeLa cells treated with NO donors showed decreased autolysosome formation. HEK293 cells expressing nitric oxide synthases demonstrated decreased LC3-II levels and autophagosome synthesis in the absence or presence of Bafilomycin A_1_. Additionally, autophagosome synthesis was increased in the L-NAME-treated mouse primary cortical neurons and HeLa cells in the presence of Bafilomycin A_1_ [177]. Overexpression of different nitric oxide synthase isoforms or treatment of the cells with NO donors resulted in impaired autophagic flux suggesting the deleterious role of NO on macroautophagy in these cells [177]. However, the effect of excessive NO generation on cardiac macroautophagy is not known. NO is an important signaling molecule for normal cardiovascular regulation, and physiological levels of NO may be important to facilitate normal macroautophagic function in the hypertensive heart.

#### 2.5.5. Macroautophagy and Diabetic Cardiomyopathy

Diabetic cardiomyopathy is characterized by metabolic dysfunction which coincides with left ventricular hypertrophy and diastolic dysfunction [178]. The main regulator of glucose homeostasis, insulin, activates mTOR in the presence of amino acids, and this results in the phosphorylation and inhibition of Atg1/ULK1 [179]. Macroautophagy inhibited by the insulin-mTOR pathway can be activated by the mTOR inhibitor rapamycin or amino acid starvation [179]. Increased autophagosome formation and increased LC3-II/LC3-I ratio suggestive of enhanced macroautophagy were observed in the hearts of mice deficient in insulin receptor substrates [180]. Consistently, a recent study demonstrated significant increases in LC3B-II and Beclin 1 levels in the hearts of the type 2 diabetic patients [181]. It may be inferred that in an effort to counteract the adverse outcome of insulin resistance/deficiency, macroautophagy machinery may be upregulated as a compensatory response to maintain normal cellular homeostasis.

The Mir30 family is highly expressed in the hearts, and Mir30c expression decreases in patients with cardiac hypertrophy [182]. Consistently, Mir30c was found to be depleted in diabetic cardiomyopathy patients. Using genetic mouse models of diabetic cardiomyopathy, the authors showed a decrease in Mir30c expression which was associated with increased Beclin 1 levels and LC3-II:LC3-I ratio [183]. Another study compared autophagic responses to diabetic cardiomyopathy in type 1 and type 2 diabetic mice both biochemically and ultrastructurally. They reported enhanced macroautophagy in type 1 diabetic heart as indicated by increased p62 levels and LC3-II/LC3-I ratio and increased activation of AMPK. On the contrary, decreased macroautophagy was observed in type 2 diabetic hearts. The ultrastructural data indicated increased lysosomes and autophagosomes in the cardiomyocytes of type 1 diabetic animal, while no mature autolysosomes or lysosomes were observed in type 2 diabetic cardiomyocytes [184]. While this study is in agreement with similar studies indicating reduced cardiac macroautophagy in type 1 diabetes [70, 180], contradictory results exist regarding the macroautophagic response in type 2 diabetic hearts [181, 183, 184].

Based on the conflicting results, the role of macroautophagy in diabetic cardiomyopathy is not clear and further investigations are warranted to determine how macroautophagy is regulated in diabetic-induced cardiac pathology. One way to resolve the differences would be to perform gain and loss of macroautophagy studies in different experimental settings of diabetic cardiomyopathy. This would further enable us to understand if macroautophagy contributes to or rescues the pathology associated with the condition.

#### 2.5.6. Macroautophagy in Doxorubicin Cardiomyopathy

Doxorubicin (DOX), also known as Adriamycin, is a chemotherapeutic agent effective against a wide range of malignancies [185]. Unfortunately, short-term or chronic use of DOX has been shown to induce cardiotoxicity [186]. Although ROS generated by DOX has been suggested to be the key mediator of its cardiotoxic effect, recent studies have suggested the involvement of macroautophagy in DOX-induced cardiotoxicity, recently reviewed by Bartlett et al. [187]. However, contradictory results exist regarding the involvement of cardiac macroautophagy response to DOX [188]. Studies have shown that DOX enhances autophagic flux and inhibition of autophagic activity rescues DOX-induced cardiotoxicity [189–191]. DOX was shown to increase LC3-II and p62 levels in the presence and absence of Bafilomycin A_1_ in neonatal cardiomyocytes which indicated increased autophagic flux [191]. Consistently, cells overexpressing GFP-LC3 showed increased autophagic vacuole formation upon DOX treatment with and without Bafilomycin A_1_. DOX-induced macroautophagic activity was associated with increased cell death [191]. Overexpression of a transcription factor, GATA4, was significantly impaired by DOX, reduced autophagic flux, and rescued DOX-induced cell death. Additionally, another study demonstrated that DOX-induced macroautophagy by upregulating the levels of Atg5 and Atg5-Atg12 complex and resveratrol reverses the DOX-induced macroautophagic activity and reduces cardiotoxicity [190]. However, more recent studies have demonstrated the protective effect of macroautophagy in DOX-induced cardiotoxicity [192–195]. In DOX-treated mice, LC3-II and p62 protein levels were increased but were not further upregulated by chloroquine treatment suggesting impaired autophagic flux [193]. Mice deficient in ultraviolet irradiation resistance-associated gene (UVRAG), an autophagy-related protein responsible for autophagosome formation and maturation, has been shown to accelerate acute as well as chronic DOX-induced cardiotoxicity. Deletion of UVRAG had an additive effect on the impaired autophagic flux in the mouse hearts treated with DOX as shown by increased LC3-II and p62 levels. This deletion was also associated with increased accumulation of ubiquitinated protein aggregates [193]. Similarly, DOX delivery was demonstrated to inhibit autophagic flux measured by decreased total LC3 levels with and without Bafilomycin A_1_ in the cardiomyocytes. This impaired autophagic flux was accompanied by the activation of Akt/mTOR pathway which negatively regulates autophagy [192]. Induction of autophagy by the mTOR inhibitor, rapamycin, attenuated DOX-induced cell death suggesting a protective role of macroautophagy in DOX-induced cardiotoxicity [192]. It was also shown that DOX treatment impaired autophagosome formation as detected by electron microscopy in mouse hearts which could be alleviated by prior starvation [194]. DOX-induced impaired cardiac autophagy was associated with decreased phosphorylation of AMPK and ULK1 which could be mitigated by prior starvation. The discrepancies in the results obtained on the role of macroautophagy in DOX-induced cardiotoxicity may be attributed to the different doses and duration of DOX treatment, as well as the different experimental models and species employed.

### 2.6. Chaperone-Mediated Autophagy and Heart Failure

A large body of evidence confirms that restoring proteasomal and macroautophagy activities can improve bulk protein degradation, which has proved to be beneficial in several models of heart failure [2, 65, 196, 197]. However, bulk protein degradation may not be ideal for the treatment of heart failure especially when the pathology arises due to a specific protein. It is therefore critical to explore new pathways and mechanisms which can specifically clear pathological proteins and likely reverse the condition. CMA is one such pathway that may be a potential solution for clearing certain proteins. CMA specifically degrades proteins bearing a KFERQ-like motif, which could be exploited for treating and preventing heart failure. Dice lab has done extensive studies in resolving the role of CMA in various tissues like the liver, kidney, lungs, and spleen with CMA being best characterized in the liver [81, 85, 198]. The contribution of CMA to various pathological conditions like nephropathies, lysosome storage disorders, and neurodegenerative diseases has been established [198]. CMA activity can be assessed by LAMP2a protein levels. Gain and loss of LAMP2a expression have been shown to be sufficient and necessary for CMA activity [85, 92]. LAMP2a expression has been detected in the hearts of fasted animals and in isolated cardiomyocytes [78, 96]. Among the known substrates of CMA that have been identified, several have known functions in the heart. Regulator of calcineurin (RCAN1) is proposed to have two CMA recognition motifs and can be degraded by CMA [199]. RCAN1 is also expressed in the cardiac tissue and is thought to confer protection to cardiomyocytes against I/R injury. Similarly, calcium regulatory protein RyR2 has also been shown to be a substrate of CMA degradation [96]. RyR2 plays an important role in excitation-contraction of the heart, and its dysfunction is associated with heart failure [200]. Myocyte enhancer factor 2D (MEF2D) which is important for cardiac development is known to be a substrate of CMA [201]. Thus, the role of CMA in the degradation of these proteins in the heart and their association with pathophysiological conditions should be studied.

Mutations in the lysosomal LAMP2 gene causes Danon disease which is inherited in an X-linked dominant fashion, eventually developing a hypertrophic cardiomyopathy [202, 203]. Danon disease is also characterized as a lysosomal glycogen storage disease [204]. It is a rare genetic disorder which predominantly causes cardiac and skeletal myopathy. The phenotypic expression of Danon disease involving mental disability may vary with the stage and severity of the disease [205]. The LAMP2 mRNA undergoes alternative splicing resulting in the generation of three isoforms: LAMP2a, LAMP2b, and LAMP2c, which have similar luminal regions but differ in their cytoplasmic and transmembrane domains [206, 207]. Earlier studies have demonstrated that of the three LAMP2 isoforms, mutation in LAMP2b was sufficient and important to cause Danon disease [203, 208, 209]. While mutations in LAMP2a lead to loss of CMA activity [141], further studies are required to delineate the involvement of LAMP2a in Danon disease. Additional studies of CMA and LAMP2a will further refine our understanding on the precise function of CMA in cardiac myopathy and its relationship to Danon disease.

A recent study found that aging increases CMA activity in the hearts indicated by increased LAMP2a levels [210]. The increase in CMA activity may be an adaptive mechanism to compensate for the loss of macroautophagy or proteasome function observed in aged animals [211, 212]. Causal studies on CMA function in the heart have yet to be published. Unraveling the contribution of CMA in protecting against cardiac pathology may enable us to therapeutically target this pathway in treating and preventing the pathology.

### 2.7. Mitophagy and Cardiovascular Diseases

A key feature of the heart is its contractile function with high energy requirements, and the major supplier of ATP is the mitochondria. The selective degradation of mitochondria by macroautophagy is termed as mitophagy [115]. Mitophagy is an important regulator of mitochondrial quality control in the heart. Dysfunctional mitochondria due to excessive reactive oxygen species generation and impaired high energy phosphate metabolism are associated with heart failure pathology [98]. Emerging evidence shows that markers of impaired mitophagy (assessed by colocalization between LC3 and mitochondria [213, 214], ubiquitination mitochondrial proteins, and binding of LC3-II to mitochondria [214]) lead to the accumulation of damaged mitochondria. Increased accumulation of dysfunctional mitochondria is associated with the progression of heart failure suggesting a protective role for higher levels of mitophagy in the heart [213–215]. The cardioprotective nature of mitophagy was further confirmed in various genetically modified mouse models [213, 216]. The mitochondrial proteins, PINK1 and PARKIN, are associated with mitophagy regulation [108, 110]. Transgenic mice overexpressing the downstream mitophagy regulator, *Parkin*, exhibited increased accumulation of mitochondria in the autophagosomes in the aged hearts. This was accompanied by improved cardiac function in the aged transgenic compared to the wild-type mouse hearts [213]. Consistently, deficiency of *parkin* exhibited a more severe cardiac phenotype which was attributed to accumulation of aberrant mitochondria and impaired mitophagy following experimental myocardial infarction [214]. The mitochondrial protein PINK1 has been shown to maintain mitochondrial homeostasis in the heart [216]. Deletion of *pink1* in mice leads to the development of progressive cardiac hypertrophy. *pink1* deficiency was accompanied by increased mitochondrial dysfunction and oxidative stress which potentiated the severity of hypertrophy [216]. Assays to directly measure mitophagy were not done to determine if suppression of *pink1* leads to impaired mitophagy in the heart [216].

Recent studies have implicated the mediator of mitochondrial fission, Dynamin-1-like gene (DRP1), as a putative regulator of mitophagy [217–219]. Cardiac-specific knockout of *drp1* exhibited a significant decline both in macroautophagy and autophagic degradation of mitochondria and induced cardiac dysfunction [217, 218]. Endogenous DRP1 was shown to regulate macroautophagy in the cardiomyocytes, and the loss of *drp1* resulted in decreased LC3-II and increased p62 levels accompanied by suppressed changes in tfLC3 transgenic mouse hearts [217]. The *drp1* knockout mice exhibited cardiac ventricular dysfunction and impaired mitochondrial function. However, the role of Drp1 in the selective degradation of mitochondria by autophagy (mitophagy) was not shown [217]. In another study, immunostaining of the *drp1* knockout hearts showed increased levels of a mitochondrial matrix protein, pyruvate dehydrogenase, surrounded by p62 and ubiquitin fluorescent signals [218]. Further studies showed that ubiquitinated mitochondria did not colocalize with the lysosomal marker, LAMP1, suggesting impaired mitophagy in the absence of *drp1*. Interestingly, the authors reported that the autophagic flux remained unaffected in the absence of *drp1*. These studies reveal the potential cardioprotective role of increased mitophagy under pathological conditions, and targeting the mitophagy pathway to enhance mitophagy may lead to improved cardiac outcome in heart failure patients.

BCL2L13 also known as BCL2-RAMBO is an outer mitochondrial protein which participates in mitochondrial fission in mammalian cells [220]. It was shown that *Bcl2-L-13* overexpression induced mitochondrial fragmentation in the absence of *drp1* suggesting that *Bcl2-L-13* acts independently of *drp1* in inducing mitochondrial fission. Both biochemical and ultrastructural analyses suggested that BCL2L13 participates in the engulfment of mitochondria within the autophagosomes or autolysosomes through its interaction with LC3 [220]. Additional studies are needed to determine how BCL2L13 is regulated in cardiac pathology and whether BCL2L13 can be a possible therapeutic target for the treatment of heart failure.

Due to technical limitations including lack of mitochondrial flux assays, caution should be taken while interpreting the results related to mitophagy studies. Further studies on the mechanism of mitophagy regulation may allow us to apply the loss or gain of function approaches and design reliable assays to evaluate the specific role of mitophagy in different types of pathologies.

## 3. Conclusion

Studies related to autophagy in the heart are continuously evolving with researchers testing new techniques and methodologies to enable progress in the field. Additional work is needed in delineating the molecular mechanisms of autophagy regulation. Genetic manipulation of autophagy components and pathways is needed to identify potential druggable targets to prevent or treat heart failure. The role of autophagy in the pathogenesis of heart failure has been studied in various experimental models but has yielded different results and interpretations depending on the models and assays employed. There is a universal agreement that the loss of macroautophagic function is detrimental to the heart. Before manipulating autophagy for the treatment of cardiovascular disease, the results from the published papers must be carefully interpreted to determine the effect of autophagy activation and inhibition in response to cardiac pathology. Another pathway of protein degradation, CMA, has remained largely unstudied in the heart. Emerging evidence suggests that many forms of heart failure are caused by the accumulation of mutant or misfolded proteins for which no targeted treatments exist. Defining the role of this pathway in the contribution to or protection against cardiac pathology may open up new therapeutic targets to treat heart failure.

## Figures and Tables

**Table 1:** The table shows the different macroautophagy-related proteins found in mammals and yeast. It also summarizes the different gain- and loss-of function models of macroautophagy which have been utilized for studying the role of macroautophagy in cardiac pathology. In this review, the nomenclature of autophagy-related genes and proteins have been adopted following “Guidelines for the Use and Interpretation of Assays for Monitoring Autophagy (3rd Edition)” [135]. ∗∗ indicates that the loss or gain of an ATG gene has not been studied in the heart. GABARAP: gamma-aminobutyrate receptor-associated protein; GABARAP: GABA type A receptor-associated protein; GABARAPL1: GABA type A receptor-associated protein like 1; GABARAPL2: GABA type A receptor-associated protein like 2; GATE-16: Golgi-associated ATPase enhancer of 16 kDa; SNX30: sorting nexin family member 30; SNX4: sorting nexin family member 4; ULK 1: unc-51-like autophagy activating kinase 1; ULK 2: unc-51-like autophagy activating kinase 2; WIPI1: WD repeat domain, phosphoinositide interacting 1; WIPI2: WD repeat domain, phosphoinositide interacting 2; WIPI3: WD repeat domain, phosphoinositide interacting 3; WIPI4: WD repeat domain, phosphoinositide interacting 4.

Mammals	Yeast	Mammalian genetic models of autophagy	Results
ULK 1	Atg1	Loss of function: Cardiac-specific *Ulk1* deletion: Myh6-cre/*ulk1^flox/flox^* Lipoprotein lipase deletion: Myh6-cre/Lpl^flox/flox^ Combined deletion: Myh6-cre/*ulk1*^flox/flox^, Lpl^flox/flox^	Potential therapeutic strategy for regulating cardiac lipoprotein lipase activity in obesity-related cardiomyopathy [73]
ULK 2			

ATG2A ATG2B	Atg2	^∗∗^	Role in cardiac pathology is not known.

ATG3	Atg3	^∗∗^	Role in cardiac pathology is not known.

ATG4A ATG4B ATG4C ATG4D	Atg4	Loss of function: knockout of rat *Atg4B* and human *ATG4B* to define the mechanism of action and tissue distribution of ATG4B	ATG4B is expressed lowly in rat hearts [221]. Its function in cardiac pathology is not known.

ATG5	—	Loss of function: tamoxifen inducible *Atg5* in *atg5^flox/flox^*: *MerCreMer*^+^ (*atg5^flox/flox^*; *Cre*^+^); and *atg5^flox/flox^*: *MerCreMer*^−^* (atg5^flox/flox^*) mice	During heart failure, increase in autophagy is a protective mechanism adapted by the heart [65].
		A dominant form of *atg5*, *atg5^K130R^*, was expressed in mouse HL-1 cells.	Autophagy plays a protective role during I/R injury in cardiomyocytes [66].

ATG6/BECN1	Atg 6/Vps30	Loss of function: *Beclin 1^+/−^* transgenic mice Gain of function: *Beclin 1* overexpression driven by the cardiomyocyte-specific *α*-MHC promoter	Autophagy contributes to the pathogenesis of pressure overload-induced heart failure [71].
		Loss of function: heterozygous deletion of *Beclin 1* (*BCN1^+/−^*) Gain of function: *BCN1* single transgenic mice and tetracycline-controlled BCN1-tTA double transgenic (DTG) mice	Loss of autophagy protective in diabetic induced cardiac injury [70]
		Loss of function: *GFP-LC3/Beclin 1*^+/−^ transgenic mice	*Beclin 1* deletion leads to loss of autophagy and is protective in I/R injury [72].

ATG7	Atg7	Gain and loss of function: CryAB^R120G^ expressing cardiomyocytes were treated with *Atg7* siRNA and *Atg7* adenovirus. Gain of function: doxycycline-controlled expression of *Atg7* in transgenic mice	*ATG7 plays an important role in ameliorating the pathology associated with CryAB^R120G^* [2, 67].
		Loss of function: tamoxifen-inducible cardiac-specific *Atg7^flox/flox^*; *Cre*	*Autophagy is protective during myocardial ischemic reperfusion* [69].

GABARAP subfamily [222] GABARAP/ATG8A GABARAPL1/GEC1/ATG8B GABARAPL2/GATE16/ATG8C	Atg8	^∗∗^	Role in cardiac pathology is not known.
LC3 subfamily [222] MAPLC3A, MAPLC3B, MAPLC3BII, MAPLC3C			LC3-II levels are used as indicators for studying autophagy [124–127].

ATG9A ATG9B	Atg9	^∗∗^	Role in cardiac pathology is not known.
ATG10	Atg10	^∗∗^	Role in cardiac pathology is not known.
Not identified	Atg11	^∗∗^	
ATG12	Atg12	^∗∗^	Role in cardiac pathology is not known.

ATG13	Atg13	Loss of function: *atg13*-deficient mice were generated using CRISPR/Cas9 system.	Loss of Atg13 causes myocardial growth defects in developing embryos [75].

ATG14/ATG14L/BAKOR	Atg14	^∗∗^	Role in cardiac pathology is not known.
Not identified	Atg15	^∗∗^	

ATG16L1 ATG16L2	Atg16	A gene trap-induced hypomorphic allele of the *Atg16L1* (*atg16L1-HM*)	Loss of autophagy is protective in diabetic-induced cardiac injury [70].

RB1CC1 (RB1-induced coiled coil 1)/FIP200	Atg17	Loss of function: *FIP200*^Δ*/*Δ^ mice caused by *FIP200* gene ablation and *FIP200^flox/flox^* were generated.	FIP200 is important for normal cardiac development, and its deletion causes embryonic lethality involving defects in the heart and liver [74].

WIPI1, WIPI2, WIPI3, WIPI4	Atg8	^∗∗^	Role in cardiac pathology is not known.

Not identified	Atg19, Atg20	___	___

WIPI2 (WD repeat domain, phosphoinositide interacting 2)	Atg21	^∗∗^	Role in cardiac pathology is not known.

Not identified	Atg22, Atg23	___	___

SNX30	Atg24A	^∗∗^	Role in cardiac pathology is not known.
SNX4	Atg24B		

Not identified	Atg25, Atg26 Atg27, Atg28 Atg29, Atg30 Atg31	___	___

BCL2L13 (BCL2-like 13)/Bcl2-RAMBO	Atg32	Loss of function: Bcl2-L-13 siRNA Gain of function: overexpression of Bcl2-L-13	The role of BCL2L13 in mitochondrial homeostasis was studied [220]. Role in cardiac pathology is not known.

Not identified	Atg33, Atg34 Atg35	___	___

ATG101	Atg101	^∗∗^	Role in cardiac pathology is not known.
